# Catecholamine up-regulates MMP-7 expression by activating AP-1 and STAT3 in gastric cancer

**DOI:** 10.1186/1476-4598-9-269

**Published:** 2010-10-12

**Authors:** Ming Shi, Dan Liu, Huijun Duan, Caili Han, Bo Wei, Lu Qian, Changguo Chen, Liang Guo, Meiru Hu, Ming Yu, Lun Song, Beifen Shen, Ning Guo

**Affiliations:** 1 Department of Molecular Immunology, Institute of Basic Medical Sciences, Beijing 100850, P.R. China; 2 Department of Pathology, Hebei Medical University, Shijiazhuang 050017, P.R. China; 3 Department of Surgery, General Hospital of PLA, Yongding Road 69, Haidian District, Beijing 100039, P.R. China

## Abstract

**Background:**

Stress, anxiety and depression can cause complex physiological and neuroendocrine changes, resulting in increased level of stress related hormone catecholamine, which may constitute a primary mechanism by which physiological factors impact gene expression in tumors. In the present study, we investigated the effects of catecholamine stimulation on MMP-7 expression in gastric cancer cells and elucidated the molecular mechanisms of the up-regulation of MMP-7 level by catecholamine through an adrenergic signaling pathway.

**Results:**

Increased MMP-7 expression was identified at both mRNA and protein levels in the gastric cancer cells in response to isoproterenol stimulation. β2-AR antigonist effectively abrogated isoproterenol-induced MMP-7 expression. The activation of STAT3 and AP-1 was prominently induced by isoproterenol stimulation and AP-1 displayed a greater efficacy than STAT3 in isoproterenol-induced MMP-7 expression. Mutagenesis of three STAT3 binding sites in MMP-7 promoter failed to repress the transactivation of MMP-7 promoter and silencing STAT3 expression was not effective in preventing isoproterenol-induced MMP-7 expression. However, isoproterenol-induced MMP-7 promoter activities were completely disappeared when the AP-1 site was mutated. STAT3 and c-Jun could physically interact and bind to the AP-1 site, implicating that the interplay of both transcriptional factors on the AP-1 site is responsible for isoproterenol-stimulated MMP-7 expression in gastric cancer cells. The expression of MMP-7 in gastric cancer tissues was found to be at the site where β2-AR was overexpressed and the levels of MMP-7 and β2-AR were the highest in the metastatic locus of gastric cancer.

**Conclusions:**

Up-regulation of MMP-7 expression through β2-AR-mediated signaling pathway is involved in invasion and metastasis of gastric cancer.

## Background

Gastric cancer is the second most common leading cause of cancer related death, with around 700 000 deaths each year [1,2]. One of the important factors in the pathogenesis of gastric cancer is chronic infection with *Helicobacter pylori *[3]. However, the majority of infected individuals do not develop malignancy and the outcome of the infection is dependent on host, environmental and other factors [4]. There is growing evidence supporting the role of psychological stress in the gastric cancer onset and development [5]. Several epidemiological studies have demonstrated that psychological or behavioral stress factors may accelerate the progression of gastric cancer [6,7]. However, the precise mechanism by which psychological stress acts in gastric cancer progression is unclear.

Stress initiates a response of the hypothalamic-pituitary-adrenal axis (HPA), resulting in increased catecholamine level [8,9]. Several studies have shown that the stimulation of catecholamine interferes with bio-behaviors of tumor cells directly, mainly through β2-adrenergic receptors (β2-AR)-mediated signaling pathway [10-14]. Recent observations pointing to a potential mechanism for the progression of ovarian, nasopharyngeal and pancreatic cancers indicate that catecholamine may modulate the expression of matrix metalloproteinase (MMP)-2 and MMP-9 by stroma and tumor cells [15-17]. It raises an interesting question that psychological stress may play a role in the development and progression of gastric cancer by modulating the expression of MMPs through β2-AR mediated signaling pathway.

Accumulated lines of evidence show that MMP-7 is involved in the invasion and metastases of gastric cancer [18-24]. MMP-7 is the smallest known member of MMP family and possesses the highest extracellular matrix (ECM)-degradative activity against a variety of ECM components, including elastin, gelatin, type IV collagen, fibronectin, vitronectin, laminin, entactin, aggrecan and proteoglycans, among the MMPs [25,26]. It is also capable of triggering the activation of an MMP cascade [27]. A unique feature of MMP-7 is its restricted expression predominantly in epithelial cells of glandular tissue including normal mammary, liver, pancreas, prostate and peribronchial glands [28].

It has been found that MMP-7 is overexpressed in the invasive cancers of digestive organs, such as oesophageal [29], gastric [30], pancreatic [31,32], colorectal [33-35], liver [36] and other organs. The expression level of MMP-7 is significantly associated with the transformation of tumor cells, phenotypes of aggressive cancers and stage of tumor progression [37], especially in tumors of gastrointestinal tract. The overexpression of MMP-7 is frequently identified in premalignant gastric lesions and most gastric carcinomas [18-21,24,38]. A positive correlation of MMP-7 level with the tumor invasion of the gastric wall, lymph node metastasis, peritoneal dissemination and survival of gastric cancer patients has been documented in several studies [23]. MMP-7 has been recognized as an important mediator of cancer progression and considered as an independent prognostic marker for primary gastric cancer. However, the possible molecular mechanisms of MMP-7 overexpression in gastric cancer are still largely unexplored.

In the present study, we investigated the effects of catecholamine stimulation on MMP-7 expression in gastric cancer cell lines and elucidated the molecular mechanisms of the up-regulation of MMP-7 level by catecholamine through an adrenergic signaling pathway.

## Results

### Isoproterenol stimulation up-regulates the expression of MMP-7

**Figure 1:**
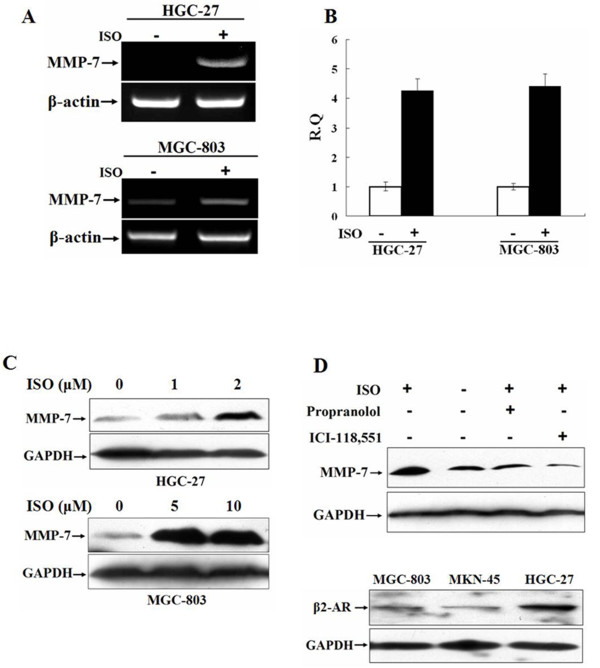
**Isoproterenol stimulation up-regulates the expression of MMP-7**. A and B, HGC-27 and MGC-803 cells were incubated overnight in serum-free medium and then treated with 2 or 10 μM isoproterenol, respectively. The expression of MMP-7 mRNA was analyzed by RT-PCR (A) and real-time PCR (B). C, HGC-27 cells were stimulated with 0, 1 or 2 μM isoproterenol and MGC-803 cells with 0, 5 and 10 μM isoproterenol after serum starvation. The expression of MMP-7 protein was analyzed by Western blot. D, MGC-803 cells were treated with 10 μM propranolol or 1 μM ICI-118,551 for 1 h and then with 10 μM isoproterenol. MMP-7 expression was analyzed by Western blot (upper panel); the expression of β2-AR in MGC-803, MKN-45 and HGC-27 cells was detected by Western blot (lower panel).

It was shown that the expression of MMP-9 in ovarian cancer was significantly enhanced in tumor associated macrophages in the patients with elevated depressive symptoms [15] and catecholamine potentiated LPS-induced expression of MMPs in human monocytes [39]. In order to investigate whether catecholamine associates with the expression of MMP-7 in gastric cancer cells, we first inspected the effects of β-AR agonist isoproterenol on the transcription of MMP-7 gene in gastric cancer cell lines HGC-27 and MGC-803. The doses of catecholamine used in this study were selected to reflect the physiologic conditions of this hormone in tumors [40,41]. Semi-quantitative and real-time PCR analyses showed that MMP-7 expression at transcriptional level was low in gastric cancer cell lines. When the cells were exposed to 10 μM of isoproterenol in the serum-free media for 12 h, MMP-7 mRNA levels were remarkably increased in both cell lines (Fig. 1A and 1B). To determine whether the increased level of MMP-7 mRNA leads to a similar increase in the protein production, MMP-7 expression in HGC-27 and MGC-803 cells was analyzed by Western blot. As shown in Fig. 1C, isoproterenol stimulation resulted in a significant up-regulation of MMP-7 expression in HGC-27 and MGC-803 cells. β2-AR, which mediates most of the effects of catecholamine, has been identified in breast and ovarian cancer cells [11,13]. To clarify the role of β2-AR-mediated signaling in MMP-7 expression, we utilized a β-AR antagonist propranolol and selective β2-AR antagonist ICI-118,551 to test if the effect of isoproterenol on MMP-7 expression can be inhibited. Our data showed that the treatment with propranolol and ICI-118,551 efficiently blocked isoproterenol-stimulated MMP-7 expression (Fig. 1D, upper panel). We then examined whether β2-AR is expressed in gastric cancer cells. The data showed that the expression of β2-AR was detectable in three gastric cancer cell lines including HGC-27, MGC-803 and MKN-45 (Fig. 1D, lower panel). These data indicated that isoproterenol stimulated the production of MMP-7 in gastric cancer cell lines and that the up-regulation of MMP-7 expression by isoproterenol was mainly triggered by β2-AR mediated signaling.

### Isoproterenol up-regulates MMP-7 promoter activity and activates STAT3 and AP-1

**Figure 2:**
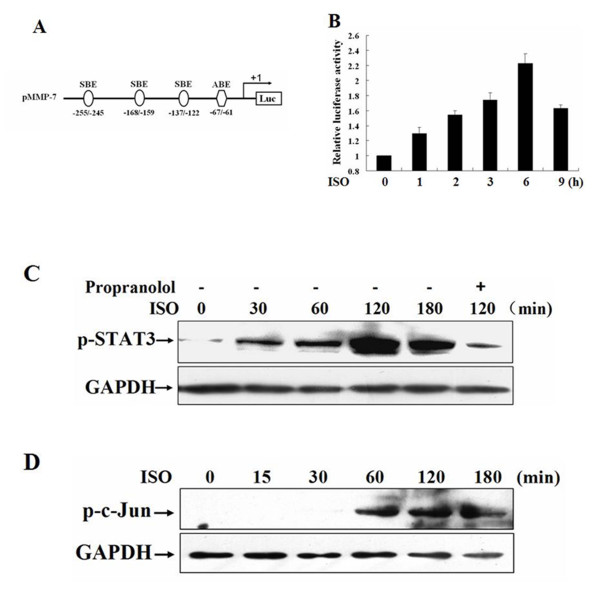
**Isoproterenol up-regulates MMP-7 promoter activity and activates STAT3 and AP-1**. A, schematic representation of the plasmid pMMP-7 containing a luciferase reporter gene driven by a 340 bp MMP-7 promoter fragment. SBE, STAT3 site; ABE, AP-1 site. B, MGC-803 cells were co-transfected with pMMP-7 and pRL-TK. After transfection for 48 h, the cells were incubated in serum-free medium for an additional 24 h and then stimulated with 10 μM isoproterenol for 0, 1, 2, 3, 6 or 9 h, respectively. MMP-7 promoter activities were assessed by luciferase assays. C and D, MGC-803 cells were treated with 10 μM propranolol for 1 h and then with 10 μM isoproterenol for 0, 15, 30, 60, 120 or 180 min, respectively. The phosphorylation of STAT3 and c-Jun was analyzed by Western blot with anti-phosphor-STAT3 and anti-phosphor-c-Jun rabbit polyclonal antibodies. ISO, isoproterenol.

To explore the molecular mechanisms by which catecholamine up-regulates MMP-7 expression, we first determined if isoproterenol stimulation can directly affect MMP-7 promoter activity. We constructed a plasmid pMMP-7 containing a luciferase reporter gene driven by a 340 bp MMP-7 promoter fragment (-296 - +44) (Fig. 2A). MGC-803 cells were transfected with pMMP-7 and the effect of isoproterenol on MMP-7 promoter activity was assessed by luciferase assays. As shown in Fig. 2B, after isoproterenol stimulation for 1 h, the luciferase activities began to rise and gradually enhanced. At 6 h after exposure, MMP-7 promoter activities showed an over twofold increase compared with the unstimulated control cells, suggesting that isoproterenol stimulation could directly induce the transactivation of MMP-7 promoter.

In our previous study [42,43], we identified an AP-1 binding site at the position -67 to -61 and three STAT3 binding sites at the positions -137 to -122, -168 to -159 and -255 to -245 in MMP-7 promoter (Fig. 2A). We also demonstrated that AP-1 and STAT3 bound to AP-1 and one of the STAT3 (-137 to -122) sites, positively regulating MMP-7 transcription. A recent study showed that catecholamine has the potential to activate STAT3. To investigate whether catecholamine stimulates MMP-7 transcription via the activation of STAT3 and AP-1, we analyzed the phosphorylation of STAT3 and c-Jun. Fig. 2C showed that isoproterenol caused the phosphorylation of STAT3 after 30 min stimulation, reaching a peak at 2 h. Although the activation of c-Jun was slow, initiated at 60 min, a maximum phosphorylation was achieved at 2 h as well (Fig. 2D). It signified that isoproterenol stimulation produced a β2-AR-mediated signal that triggered the activation of STAT3 and AP-1.

### STAT3 element in MMP-7 promoter is not required and STAT3 not sufficient for isoproterenol-induced MMP-7 expression

**Figure 3:**
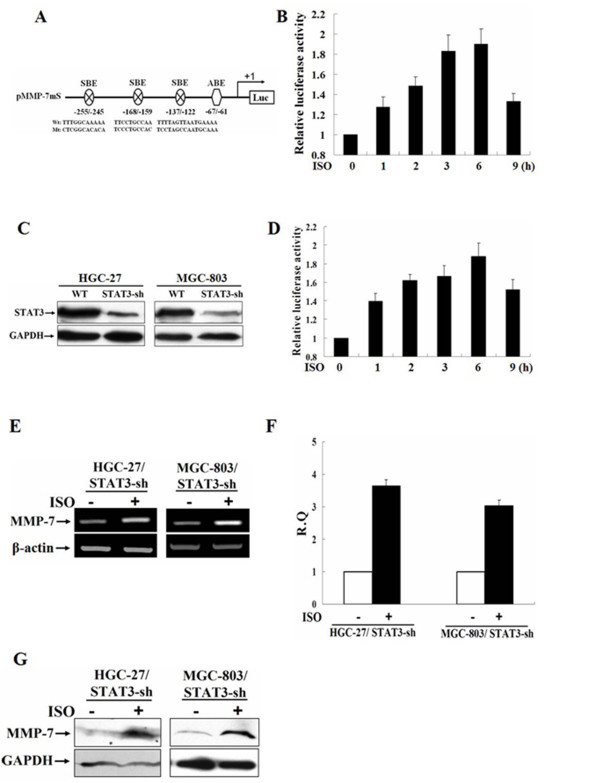
**STAT3 element in MMP-7 promoter is not required and STAT3 not sufficient for isoproterenol-induced MMP-7 expression**. A, schematic representation of the plasmid pMMP-7mS containing three mutated STAT3 sites at the positions -255 to -245, -168 to -159 and -137 to -122. B, MGC-803 cells were co-transfected with pMMP-7mS and pRL-TK, starved, and then stimulated with 10 μM isoproterenol for 0, 1, 2, 3, 6 or 9 h, respectively. MMP-7 promoter activities were assessed by luciferase assays. C, HGC-27 and MGC-803 cells were stably transfected with pGeneSuppressor expressing STAT3 shRNA. The expression of STAT3 was analyzed by Western blot in HGC-27/STAT3-sh and MGC-803/STAT3-sh cells. D, MMP-7 promoter activities were assayed in MGC-803/STAT3-sh cells after stimulation with 10 μM isoproterenol for 0, 1, 2, 3, 6 or 9 h, respectively. E through G, HGC-27/STAT3-sh and MGC-803/STAT3-sh cells were stimulated with 2 μM or 10 μM isoproterenol, respectively. The expression of MMP-7 was analyzed by RT-PCR (E), real-time PCR (F) and Western blot (G).

In order to determine whether the STAT3 elements are functional in isoproterenol-induced MMP-7 gene transcription, we first undertook the mutagenesis of three STAT3 sites through mutating the core-sequences of STAT3 sites based on pMMP-7 (Fig. 3A) and constructed the plasmid pMMP-7mS. The plasmid was transfected into MGC-803 cells. After transfection for 48 h, the cells were incubated in serum-free medium overnight and then stimulated with 10 μM of isoproterenol for 6 h. We noticed, surprisingly, that the mutation of all three STAT3 binding sites had no prominent effect on the transactivation of MMP-7 promoter. As shown in Fig. 3B, luciferase activities were gradually increased and approached a peak at 6 h after exposure. The result was very similar to that observed in the cells transfected with wild type MMP-7 promoter. To identify the role of STAT3 in this event, HGC-27 and MGC-803 cells were transfected with pGeneSuppressor expressing shRNA targeting STAT3 and HGC-27/STAT3-sh and MGC-803/STAT3-sh cells established. Fig. 3C showed that the expression of STAT3 was efficiently suppressed in both cells. Consistent with above data, isoproterenol-induced MMP-7 promoter activities appeared not to be significantly impaired in MGC-803/STAT3-sh cells (Fig. 3D). Furthermore, isoproterenol-induced MMP-7 expression at both mRNA and protein levels was not importantly affected by silencing STAT3 expression as demonstrated by RT-PCR, real-time PCR and Western blot (Fig. 3E, F and 3G). The results suggested that STAT3 element in MMP-7 promoter is not required and STAT3 not sufficient for isoproterenol-induced MMP-7 expression.

### AP-1 plays a critical role in isoproterenol-induced MMP-7 expression

**Figure 4:**
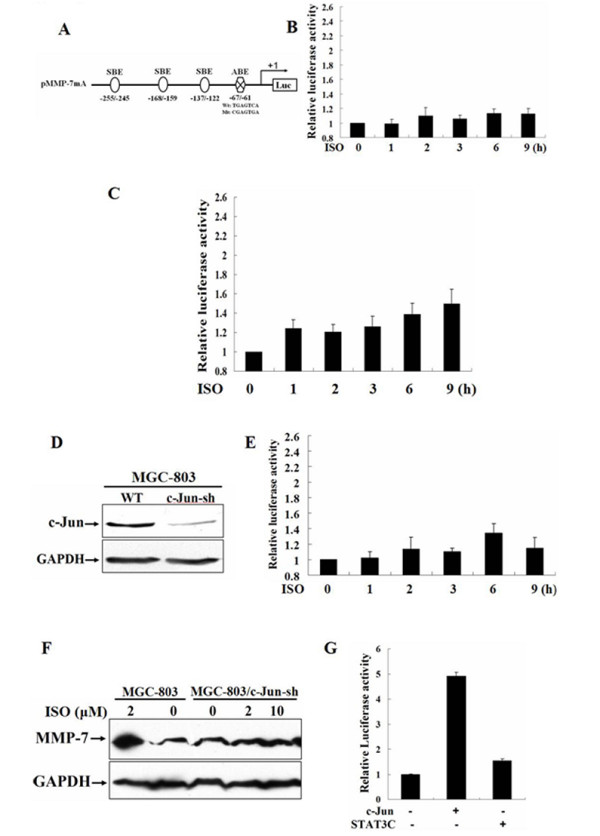
**AP-1 plays a critical role in isoproterenol-induced MMP-7 expression**. A, schematic representation of the plasmid pMMP-7mA containing a mutated AP-1 site at the position -67 to -61. B, MGC-803 cells were co-transfected with pMMP-7mA and pRL-TK, starved and then stimulated with 10 μM isoproterenol for 0, 1, 2, 3, 6 or 9 h, respectively. MMP-7 promoter activities were assessed by luciferase assays. C, MGC-803 cells co-transfected with TAM67, pMMP-7 and pRL-TK were induced with 10 μM isoproterenol. MMP-7 promoter activities were analyzed by luciferase assays. D, MGC-803 cells stably expressing shRNA targeting c-Jun (MGC-803/c-Jun-sh) were established and c-Jun expression was analyzed by Western blot. E, MMP-7 promoter activities were assayed in MGC-803/c-Jun-sh cells after stimulation with 10 μM isoproterenol for 0, 1, 2, 3, 6 or 9 h, respectively. F, MMP-7 expression was analyzed in MGC-803/c-Jun-sh cells after stimulation with 0, 2 or 10 μM isoproterenol, respectively. G, the plasmids expressing c-Jun and STAT3C were transfected into MGC-803/c-Jun-sh cells, respectively. MMP-7 promoter activities were analyzed by luciferase assays.

The above data was unexpected, since STAT3 could be activated by isoproterenol stimulation. In addition, in our previous study [42,43], we demonstrated that STAT3 and AP-1 participated in regulating Her2 signaling-mediated MMP-7 expression in breast cancer cells. We then sought to investigate whether AP-1 influences isoproterenol-induced MMP-7 expression. MGC-803 cells were transfected with the plasmid pMMP-7mA containing a mutated AP-1 site at the position -67 to -61 in MMP-7 promoter and luciferase activities measured (Fig. 4A). Surprisingly, isoproterenol-induced MMP-7 promoter activities were completely disappeared at all time points (Fig. 4B), suggesting that the AP-1 site was crucial in isoproterenol-induced MMP-7 expression.

To confirm isoproterenol-induced MMP-7 expression is controlled by AP-1, we then examined whether the transactivation of MMP-7 promoter induced by isoproterenol could be inhibited by a dominant negative c-Jun mutant TAM67, which lacks the c-Jun 5'-transactivating domain but possesses a functional c-Jun leucine zipper and DNA-binding domain. After transient transfection with TAM67 into MGC-803 cells, isoproterenol-induced transactivation of MMP-7 promoter was analyzed by luciferase assays. As shown in Fig. 4C, the expression of the dominant negative c-Jun exhibited a conspicuous dampening impact on MMP-7 promoter activities. To further verify the critical role of AP-1, we tested whether blocking endogenously expressed c-Jun can inhibit the expression of MMP-7. The MGC-803/c-Jun-sh cells were established (Fig. 4D) and MMP-7 promoter activities analyzed by luciferase assays. The data in Fig. 4E showed that the activities of MMP-7 promoter were strikingly reduced by knock-down of c-Jun expression. Western blot analysis also demonstrated that isoprotereol-induced MMP-7 expression was markedly decreased in MGC-803/c-Jun-sh cells, whereas a substantially large amount of MMP-7 protein was detected in the parental cells after isoproterenol stimulation (Fig. 4F). Notably, the overexpression of exogenous c-Jun dramatically restored MMP-7 promoter activities in MGC-803/c-Jun-sh cells, but constitutively activated STAT3 mutant STAT3C only minimally activated MMP-7 promoter when c-Jun expression was silenced (Fig. 4G). These data proved that AP-1 dominated isoproterenol-induced MMP-7 expression.

### c-Jun and STAT3 synergistically regulate MMP-7 expression in response to isoproterenol stimulation

Cooperation of Stat3 and c-Jun in regulating a variety of gene transcription has been reported. Our previous study demonstrates that the activation of MMP-9 promoter is dependent upon the interaction of Stat3 and AP-1 [44]. As mentioned above, isoproterenol stimulation induced the activation of STAT3 and AP-1 simultaneously. We speculated that Stat3 and AP-1 may synergistically participate in the regulation of isoproterenol-stimulated MMP-7 expression. It has been indicated that AP-1 cooperates with STAT3 in interleukin 6-induced transactivation of the IL-6 response element in the absence of direct AP-1 DNA binding [45]. It prompted us to test the possible cooperative binding of AP-1/STAT3 to the AP-1 site.

**Figure 5:**
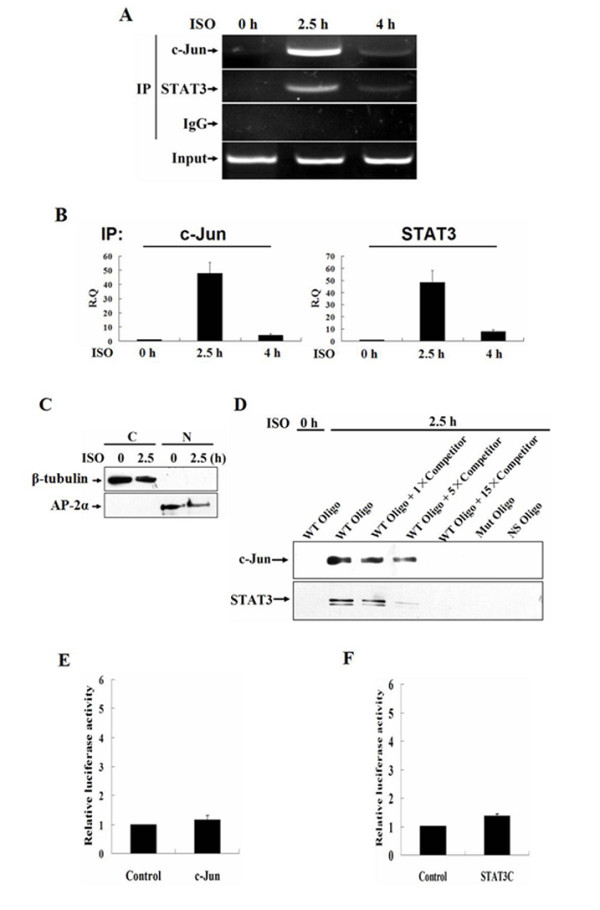
**c-Jun and STAT3 synergistically regulate MMP-7 expression**. A and B, MGC-803 cells were stimulated with 10 μM of isoproterenol for 0, 2.5 or 4 h, respectively. The chromatin DNA/nuclear protein complexes were prepared. Binding of STAT3 and c-Jun to the AP-1 site was analyzed by the immunoprecipitation with anti-STAT3 and anti-AP-1 antibodies followed by PCR (A) and real-time PCR (B). C, MGC-803 cells were treated with 10 μM isoproterenol for 0 or 2.5 h, respectively. The nuclear extract was prepared with a Nuclear-Cytosol Extraction Kit. β-tubulin and AP-2α were analyzed by Western blot to examine the separation of cytoplasmic and nuclear proteins. C, cytosolic proteins; N, nuclear proteins. D, 200 μg of the nuclear extracts was incubated at 4°C for 4 h with the biotinylated oligonucleotides containing AP-1 consensus sequence previously coupled to Dynabeads M-280 in the presence or absence of one, five or 15 fold amount of the double-stranded oligonucleotide competitors. Incubation of the nuclear proteins with the double-stranded oligonucleotides containing a mutated AP-1 site (Mut Oligo) or non-specific double-stranded oligonucleotides (NS Oligo) was used as controls. The protein/DNA complexes were separated with a Dynal magnet, and subjected to SDS-PAGE. STAT3 and c-Jun were detected by Western blot with anti-STAT3 and anti-c-Jun antibodies. E and F, MGC-803 cells were co-transfected with the plasmids pMMP-7mA and pCDNA3.1/c-Jun or STAT3C and luciferase activities were assayed.

To determine whether that STAT3 and AP-1 can be recruited to the AP-1 site in MMP-7 promoter in vivo, MGC-803 cells were stimulated with 10 μM of isoproterenol for 0, 2.5 or 4 h, respectively. The chromatin DNA/nuclear protein complexes were prepared and binding of STAT3 and c-Jun to the AP-1 site was analyzed by ChIP assays. The PCR product selected for amplification extends from the -76 to +165 region harboring the AP-1 site in MMP-7 promoter. The immunoprecipitation with anti-STAT3 and anti-c-Jun antibodies followed by PCR yielded an expected 241 bp band from the precipitants after isoproterenol stimulation for 2.5 h. The intensities of these bands were weakened after 4 h. In contrast, in unstimulated control cells no binding of STAT3 to this site was observed and the binding of c-Jun barely detectable. No band was amplified by immunoprecipitation using rabbit IgG (Fig. 5A). The data were confirmed by real-time PCR (Fig. 5B). These data demonstrated that the association of STAT3 and c-Jun with MMP-7 promoter *in vivo *was specific and that isoproterenol stimulation induced binding of STAT3/c-Jun to the AP-1 element.

To further characterize the interaction of STAT3/c-Jun with the AP-1 site, we performed a DNA affinity precipitation assay. After MGC-803 cells were treated with isoproterenol for 2.5 h, the nuclear extract was prepared and its quality was assessed (Fig. 5C). The biotinylated oligonucleotides corresponding to -74 to -52 region of MMP-7 promoter were incubated with 200 μg of the nuclear extracts for the pull-down assays. The streptavidin coated magnetic beads were used to precipitate biotin-labeled double-stranded oligonucleotides and associated DNA binding proteins. The binding of the nuclear proteins to the biotinylated oligonucleotides was analyzed in the presence of one, five, or 15 fold amount of double-stranded oligonucleotide competitors containing AP-1 consensus sequences. Incubation of the nuclear proteins with the double-stranded oligonucleotides containing a mutated AP-1 site or non-specific double-stranded oligonucleotides was used as controls. The resulting DNA-protein complexes were resolved by SDS-PAGE, followed by Western blot with anti-c-Jun and anti-STAT3 antibodies. The association of both transcriptional factors with the oligonucleotides containing AP-1 consensus sequences could be clearly detected in the nuclear extracts from isoproterenol-stimulated cells, but not from unstimulated cells. Competition with fifteen fold amount of the competitors utterly abolished the formation of the biotinylated DNA/protein complexes (Fig. 5D). No binding of c-Jun and STAT3 to either the oligonucleotides containing a mutated AP-1 site or nonspecific oligonucleotides was detected (Fig. 5D). This experiment provides in vitro evidence to confirm that STAT3 and c-Jun, under catecholamine stimulation, can physically interact and bind to the AP-1 site to achieve transactivation of MMP-7 gene in gastric cancer cells.

We showed that isoproterenol-induced MMP-7 promoter activation was not disrupted by the mutagenesis of the core-sequences of all three STAT3 sites, suggesting that the STAT3 elements may not act in isoproterenol-induced MMP-7 gene transcription in gastric cancer cells. To further verify the role of AP-1 element, the plasmid pMMP-7mA was co-transfected into MGC-803 cells with either pCDNA3.1/c-Jun or STAT3C, respectively. Interestingly, the mutation of AP-1 binding site thoroughly blocked the activation of MMP-7 promoter in the transfected cells overexpressing either c-Jun or STAT3C (Fig. 5E and 5F), indicating that the AP-1 site is an important regulatory element and the synergistic regulation of isoproterenol-induced MMP-7 expression by STAT3 and AP-1 relies on this element.

### β2-AR and MMP-7 were colocalized in gastric cancer tissues

**Figure 6:**
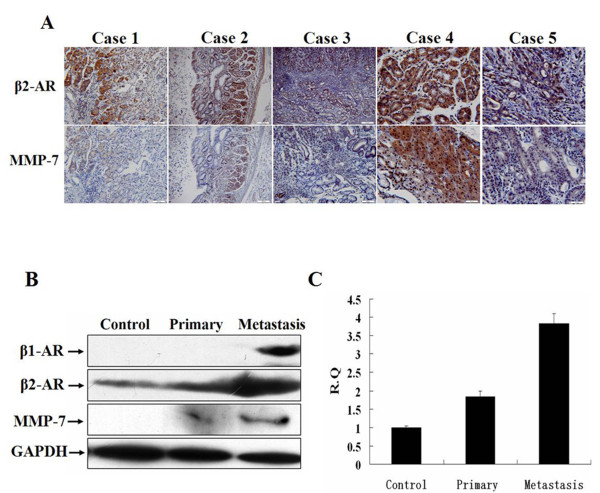
**β2-AR and MMP-7 were overexpressed in gastric cancer tissues**. A, five gastric cancer tissue samples were collected. The expression of β2-AR and MMP-7 was analyzed by immunohistochemistry. B, the expression of MMP-7, β1-AR and β2-AR in the cancerous, peri-cancerous and metastatic tissues was analyzed by Western blot. C, the level of MMP-7 mRNA in the cancerous, peri-cancerous and metastatic tissues was analyzed by real-time PCR.

The overexpression of MMP-7 was frequently at the invasive front of human gastric cancer and directly associated with prognosis in patients with gastric cancer. To study the correlation of β2-AR level with MMP-7 expression in human gastric cancer, we collected five gastric cancer tissue samples and analyzed the expression of β2-AR and MMP-7 by immunohistochemical labeling. Interestingly, the strong expression of β2-AR and MMP-7 emerged at the same region of the tumor tissues (Fig. 6A), suggesting an intimate relationship between MMP-7 and β2-AR. To further investigate the role of MMP-7 and β2-AR in the metastasis of gastric cancer, we analyzed the expression MMP-7 and β-ARs in the cancerous, peri-cancerous and metastatic tissues from a patient with gastric cancer. As shown in Fig. 6B, the expression of β1-AR, β2-AR and MMP-7 at protein level was higher in cancerous tissue than in peri-cancerous tissue. However, the highest expression of them was found in the metastatic tissue. Moreover, MMP-7 mRNA was also overexpressed in the metastatic tissues (Fig. 6C). These data strongly imply a close link among MMP-7, β2-AR and metastasis of gastric cancer.

## Discussion

The present study was based on the hypothesis that psychosocial stress may be a predisposing factor in gastric cancer. Stress, anxiety and depression can cause complex physiological and neuroendocrine changes that influence multiple systems, including the digestive system. The cancer patients often experience substantial emotional distress. The association of physiological stress with elevation of gastric acid secretion and stomachache has long been noticed [46,47]. It has been reported that chronic stress can aggravate stomach ulcers. In a recent population-based survey enrolled 2014 subjects, stress was regarded as the most powerful risk factor in gastric cancer [48]. The findings from experimental studies suggest that increased activity of the sympathetic nervous system may constitute a primary mechanism by which physiological factors impact gene expression in tumors [49]. To date, most studies investigating the mechanisms connecting stress and cancer progression has been related to indirect effects through the immunosuppression [8]. In several recent studies, stress related proteins and pathways were linked directly to the behavioral alteration of malignant cells [10-17]. However, there is a paucity of data delineating the molecular mechanisms involved.

In the present study, we identified increased MMP-7 expression in the gastric cancer cell lines in response to the stimulation of stress-related hormone catecholamine. It is estimated that the concentration of catecholamine could be over 100 times higher in the tumor microenvironment than in normal tissues and circulating plasma. MMP-7 is unique in its restricted expression in tumor cells, indicating that MMP-7 expression is in a tumor-associated fashion. We noticed that isoproterenol stimulation significantly up-regulated MMP-7 expression at both mRNA and protein levels in gastric cancer cells. β2-AR antigonist effectively abrogated isoproterenol-induced MMP-7 expression up-regulation, suggesting that β2-AR-mediated pathway is involved in the process.

MMP-7 expression is tightly controlled at the level of transcription. Our previous study demonstrated that heregulin-β-induced MMP-7 expression was regulated by HER2-mediated STAT3 and AP-1 activation in human breast cancer cell lines [42,43]. We also identified the functional AP-1 and STAT3 binding sites in the first 350 bp of the transcription start site in human MMP-7 promoter [42,43]. Another study indicated that FGF-2 could directly upregulate MMP-7 gene expression in human tumor cell lines and umbilical vein endothelial cells through AP-1 and STAT3 [50]. Accumulating evidence strongly supports that STAT3 serves as a central regulatory node on which multiple oncogenic signaling pathways converge [51]. Aberrant activation of STAT3 has been proved to play a critical role in gastric cancer development [52,53]. A recent study and our data uncovered the association of catacholamine with STAT3 activation in ovarian and breast cancer cells [12,13]. In the present study, we demonstrated that isoproterenol stimulation prominently induced the activation of STAT3 in gastric cancer cells, implicating that catecholamine may accelerate the malignant progression of gastric cancer. Our unpublished data also proved that isoproterenol stimulated the expression of MMP-2 and MMP-9 in gastric cancer cells mainly through activating STAT3. However, in this study, we found that AP-1 displayed a greater efficacy than STAT3 in isoproterenol-induced MMP-7 gene transcription and the STAT3 elements were non-functional, as silencing STAT3 expression was not effective in preventing isoproterenol-induced MMP-7 expression and mutagenesis of three STAT3 binding sites failed to repress the transactivation of MMP-7 promoter in response to isoproterenol induction. In contrast, the AP-1 site and c-Jun governed this process. Interestingly, STAT3 and c-Jun were found to bind to the single AP-1 site in MMP-7 promoter simultaneously, implicating that the interplay of both transcriptional factors at one binding site is responsible for isoproterenol-stimulated MMP-7 expression in gastric cancer cells. We have demonstrated that STAT3 and AP-1 are important transcriptional regulators of MMP-7 and MMP-9 in human breast cancer cells previously and STAT3 and AP-1 exert transcriptional regulation functions by binding to their respective elements [42-44]. Given the results of these data, catecholamine-induced MMP-7 expression may be primarily in a cell type specific manner.

Several lines of evidence indicate that the expression of MMP-7 is associated with advanced clinicopathological stages and unfavorable prognosis in gastric cancer [18-24,38,54]. Recent studies showed that MMP-7 expression was selectively up-regulated by pathogenic strains of Helicobacter pylori in Helicobacter pylori gastritis [20], which is considered as the initial stage in the progression to gastric carcinoma. MMP-7 is also identified as a target of gastrin in hypergastrinemia that is associated with gastric cancer [38]. These data suggest that MMP-7 is an important mediator in gastric cancer development. MMP-7 affects the gastric microenvironment by degrading ECM components. It also plays a key role in the activation, degradation, release and shedding of cell-surface molecules, such as pro-heparin-binding epidermal growth factor, Fas ligand, a stimulator of the death receptor FAS, cell adhesion molecule E-cadherin and insulin-like growth factor binding protein, thus stimulating cellular proliferation, inhibiting apoptosis and promoting tissue invasion and metastasis [37]. We noticed that the overexpression of MMP-7 emerged at the region where β2-AR was expressed highly and the level of MMP-7 and β2-AR is the highest in the metastatic locus of gastric cancer, suggesting a direct role of β2-AR-mediated MMP-7 expression in the invasion and metastasis of gastric cancer, and also implying an important effect of neuroendocrine "macroenvironment" on the gastric tumor microenvironment.

## Conclusions

The main findings of this study strongly support the hypothesis that up-regulation of MMP-7 expression through β2-AR-mediated signaling pathway is involved in invasion and metastasis of gastric cancer. β2-AR may serve as a target of therapeutic intervention. An understanding of how MMP-7 gene is up-regulated under stress will help to elucidate the mechanisms of MMP-7 overexpression in gastric cancer, especially at an early stage. Further identification of the down-stream effector molecules of β2-AR cascades will not only provide a more definite knowledge of the signaling network in response to catecholamine stimulation, but also close an important gap linking psychosocial stress and the cellular consequences in gastric cancer.

## Methods

### Cell culture and treatment

Human gastric cancer cell lines HGC-27 and MGC-803 (the American Type Culture Collection) were incubated in Dulbecco's modified Eagle's medium (DMEM) (Invitrogen) supplemented with 10% fetal bovine serum (FBS) (Invitrogen) at 37°C in a humidified atmosphere of 5% CO2. For the treatment with β-AR agonists, the cells were incubated overnight in serum-free medium supplemented with 0.1% BSA, 10 mM HEPES (pH 7.4) prior to stimulation. Then HGC-27 cells were treated with 0, 1, or 2 μM isoproterenol (Sigma) and MGC-803 cells 1, 5, or 10 μM isoproterenol for indicated time points. For β-AR antagonist treatment, the cells were first treated with 10 μM propranolol (TOCRIS) or 1 μM ICI-118,551 (TOCRIS) for 1 h before AR agonist stimulation.

### Constructs

The promoter region (nucleotides -296 to +44 relative to the ATG initiation codon) of human MMP-7 gene was PCR amplified from MGC-803 cell genomic DNA using the primers P1 and P2 (Table 1) with Power Pfu DNA polymerase (BioTeke) and inserted into the *Kpn *I and *Hin*d III sites immediately upstream of a firefly luciferase gene in pGL3-Basic reporter vector (Promega) designated pMMP-7. Using pMMP-7 as a templet, two mutated MMP-7 promoter fragments were generated by PCR with the primers P1 - P7 or P1, P2, P8 and P9 (Table 1), respectively, and cloned into the plasmid pGL3-Basic. The resultant recombinant plasmids were named pMMP-7mS containing three mutated STAT3 sites at the positions -255 to -245, -168 to -159, and -137 to -122 and pMMP-7mA harboring a mutated AP-1 site at the position -67 to -61. All expression constructs were verified by sequence analysis. The plasmid pRc/CMV-Stat3C-Flag (constitutive activated Stat3, Stat3C) and STAT3 dominant negative construct (STAT3-YF) are generous gifts from Drs. Bromberg and Darnell. The plasmid pCDNA3.1/c-Jun was constructed in our previous study [55]. The plasmid pRL-TK (Promega) was used as a control plasmid. The sequences 5'-GCAGCAGCTGAACAACATGTC-3' in STAT3 mRNA and 5'-AACAGGTGGCACAGCTTAAAC-3' in c-Jun mRNA were selected through the siRNA Target Finder (http://www.ambion.com/techlib/misc/siRNA_finder.html, Ambion) as siRNA target sites. The synthetic double-strand oligonucleotides (sense: 5'-TCGAGCAGCAGCTGAACAACATGTCGGAATTCGGACATGTTGTTCAGCTGCTGCTTTTT-3' and antisense: 5'-CTAGAAAAAGCAGCAGCTGAACAACATGTCCGAATTCCGACATGTTGTTCAGCTGCTGC-3' for STAT3 and sense: 5'-TCGAAACAGGTGGCACAGCTTAAACGGAATTCGGTTTAAGCTGTGCCACCTGTTTTTTT-3' and antisense: 5'-CTAGAAAAAAACAGGTGGCACAGCTTAAACCGAATTCCGTTTAAGCTGTGCCACCTGTT-3' for c-Jun) were inserted into pSuppressorNeo plasmid (Imgenex) according to the manufacturer's recommendation. The plasmid containing the corresponding scrambled target sequences was used as a control.

**Table 1:** The primers used

No.	Sequences
P1	5'-CGGGGTACCATAATGTCCTGAATGATACC-3'
P2	5'-CCCAAGCTTTGCCGTCCAGAGACAATTG-3'
P3	5'-CGGGGTACCATAATGTCCTGAATGATACCTATGAGAGCAGTCATTTGACGCTGGCAAAA-3'
P4	5'-CATTGTGTGCTCCCTGCCACTAACGATGTAATACTT-3'
P5	5'-TTGTCTTTCAAAGGATT-3'
P6	5'-TACTTCCTCGTCCTAGCCAATGCAAAATAACACATAC-3'
P7	5' - GTTATTGGCAGGAAGCACAC - 3'
P8	5'-GAAAACACTCAAACGAGTGACCTATTTCCACAT-3'
P9	5'-TTTCTTTTTAGAGTCTACAG-3'
P10	5'-GAGTGCCAGATGTTGCAGAA-3'
P11	5'-GTGAGCATCTCCTCCGAGAC-3'
P12	5'-GTGGGGCGCCCCAGGCACCA-3'
P13	5'-CTTCCTTAATGTCACGCACGATTTC-3'
P14	5'-ACACATACTTTCAAAGTTCTGTAGACTCT-3'
P15	5'-ACGGTGAGTCGCATAGCT-3'

### Transfection and luciferase assays

Cells were co-transfected with pMMP-7 or pMMP-7mS or pMMP-7mA and pRL-TK reporter plasmid containing the Renilla luciferase reporter gene using Lipofectamine 2000 (Invitrogen) according to the manufacturer's protocol. After transfection for 48 h, the cells were incubated in serum-free medium for an additional 24 h and then stimulated with 10 μM isoproterenol for indicated time points. For luciferase assays, cells were lysed in lysis buffer (Promega). Firefly and Renilla luciferase activities were measured with a dual luciferase assay kit (Promega) according to the manufacturer's instructions. All transfections were carried out in triplicate and repeated at least three times.

### Western blot

The whole cell lysates were prepared, separated by SDS-PAGE and transferred to PVDF membranes. After blocking, blots were probed with the appropriate primary antibodies overnight at 4°C. The antibodies used include anti-β2-AR rabbit polyclonal antibody (Santa Cruz Biotechnology Inc.), anti-MMP-7 mouse monoclonal antibody (Santa Cruz Biotechnology Inc.), anti-STAT3 rabbit polyclonal antibody (Cell Signaling Technology Inc.), anti-phosphor-STAT3 (Tyr705) rabbit polyclonal antibody (Cell Signaling Technology Inc.), anti-phosphor-c-Jun (Ser73) rabbit polyclonal antibody (Cell Signaling Technology Inc.), anti-β2-AR rabbit polyclonal antibody (Santa Cruz Biotechnology Inc.), and anti-glyceraldehyde-3-phosphate dehydrogenase (GAPDH) rabbit monoclonal antibody (Cell Signaling Technology Inc.). The blots were then washed and incubated with horseradish peroxidase-conjugated secondary antibodies. Bands were detected by enhanced chemiluminesence (Pierce).

### Conventional and real-time RT-PCR

For the examination of MMP-7 transcription induced by catecholamine, HGC-27 and MGC-803 cells were treated with 2 or 10 μM of isoproterenol for 12 h. Then, total RNA was isolated from HGC-27 and MGC-803 cells using TRIzol reagent (Invitrogen) following the manufacturer's instructions and quantified by spectrophotometry. cDNA was synthesized by reverse transcription kit (BioTeke) in accordance with the manufacturer's instructions and amplified by PCR with the specific primers P10 and P11 (Table 1) to screen the mRNA expression of MMP-7. The PCR products were electrophoresed on 1.0% agarose gel. Amplification of β-actin gene using the specific primers P12 and P13 (Table 1) was used as an internal control. Real-time PCR was performed using SYBR Green Supermix (TransGen Biotech) on Real-Time PCR Detection System (Eppendorf) as recommended by the manufacturer. The results were analyzed using the comparative threshold cycle method with β-actin as an internal control.

### Immunohistochemistry

Paraffin-embedded tissues were cut (~5 μm). The sections were dewaxed in xylene, and gradually hydrated in a decreasing ethanol series ending in distilled water. Endogenous peroxidase activity was quenched using 3% hydrogen peroxide in distilled water and then washed in phosphate-buffered saline (PBS). After antigen retrieval, the sections were incubated with anti-MMP-7 (Santa Cruz Biotechnology Inc.) and anti-β2-AR (Abcam) rabbit polyclonal antibodies. Following washing with PBS, the sections were subsequently incubated with horseradish peroxidase-conjugated anti-rabbit antibody (Gene Tech Biotechnology Co.). The color was developed by incubation with 3, 3'-diaminobenzidine solution. The sections were then counterstained with hematoxylin, dehydrated, and mounted. Omission of the primary antibody and substitution by non-specific rabbit IgG at the same concentration were used as negative controls.

### Chromatin immunoprecipitation (ChIP)

MGC-803 cells were treated with 10 μM of isoproterenol for 0, 2.5 and 4 h under serum-free conditions after overnight starvation. The chromatin DNA of the cells was prepared and ChIP assay performed by using the SimpleChIPTM Enzymatic Chromatin IP Kit (Cell Signaling Technology Inc.) following the protocol supplied by the manufacturer. STAT3, c-Jun, and DNA complexes were precipitated either by anti-STAT3 antibody and anti-c-Jun antibody or by rabbit IgG as the negative control. Precipitated DNA was amplified by PCR with the primers P14 and P15 (Table 1) flanking AP-1 binding site (-67 to -61) in MMP-7 promoter. Final products were resolved on a 1% agarose gel.

### Preparation of nuclear extracts and oligonucleotide pull-down assays

The 5'-biotinylated double-stranded oligonucleotides (5'-ACTCAAATGAGTCACCTATTTCC-3' and 5'-GGAAATAGGTGACTCATTTGAGT-3') corresponding to the positions -74 to -52 of MMP-7 promoter harboring AP-1 motif were synthesized by Invitrogen Biotechnology. The same double-stranded sequences that are not biotinylated were used as the competitors. The biotinylated oligonucleotides containing the mutated AP-1 binding site (5'-ACTCAAACGAGTGACCTATTTCC-3' and 5'-GGAAATAGGTCACTCGTTTGAGT-3'), in which conserved nucleotides of AP-1 consensus sequence were replaced, and the biotinylated oligonucleotides (5'-ACCAATGCAGCCCTACCTGTAGC-3' and 5'-GCTACAGGTAGGGCTGCATTGGT-3') corresponding to the positions -908 to -885 of MMP-7 promoter lacking the AP-1 binding site were also synthesized. MGC-803 cells were treated with 10 μM of isoproterenol for 0 or 2.5 h under serum-free conditions after overnight starvation. The nuclear extracts were prepared by using a Nuclear-Cytosol Extraction Kit (Applygen Technologies) according to the manufacturer's instructions. 200 μg of the nuclear extracts was incubated at 4°C for 4 h with each pair of the oligonucleotides previously coupled to Dynabeads M-280 (Invitrogen). The protein/DNA complexes were separated with a Dynal magnet, denatured in SDS sample buffer and subjected to SDS-PAGE. STAT3 and c-Jun were detected by Western blot with anti-STAT3 and anti-cJun antibodies.

### Clinical samples

All clinical tissue samples were obtained from General Hospital of PLA with the informed consent of patients and with approval for experiments from General Hospital of PLA. The peri-cancerous, cancerous and peritoneal metastatic tissues samples were obtained from a patient (male, 62 years old) diagnosed as advanced gastric cancer during radical subtotal gastrectomy.

## Competing interests

The authors declare that they have no competing interests.

## Authors' contributions

MS and DL designed study, performed experiments, and drafted manuscript, HD provided coordination, CH performed pathological examination, BW and LQ performed immunohistochemical examination, CC and LG performed ChIP assays, MH performed real-time PCR, MY constructed plasmids, LS participated in plasmid construction, BS provided grant support, NG provided grant support, coordination, and wrote manuscript. All authors have read and approved the final manuscript.
